# Development and validation of a risk score heatmap for in-hospital adverse events in older adults with acute myocardial infarction

**DOI:** 10.3389/fmed.2026.1828310

**Published:** 2026-05-04

**Authors:** Zizhu Lian, Yue Xu, Shikang Liu, Xiaohong Yang

**Affiliations:** 1 Department of Cardiovascular Surgery, The First Affiliated Hospital of Xi’an Jiaotong University, Xi’an, China; 2 Xi’an Jiaotong University Health Science Center, Xi’an, China; 3 Department of Cardiovascular Medicine, The First Affiliated Hospital of Xi’an Jiaotong University, Xi’an, China

**Keywords:** acute myocardial infarction, in-hospital adverse events, older adults, prediction model, risk score

## Abstract

**Background:**

Older adults with acute myocardial infarction (AMI) represent a particularly high-risk population for early in-hospital deterioration. Life-threatening complications—including sustained ventricular tachycardia or fibrillation (sVT/VF), cardiogenic shock (CS), and acute heart failure (AHF)—are major contributors to in-hospital adverse events (IAE), as these events may herald an increased risk of poor post-discharge prognosis. However, a simple and visually intuitive tool for early risk stratification in this population remains lacking.

**Methods:**

We conducted a retrospective cohort study of consecutive patients aged >60 years with AMI admitted between January 2019 and December 2024. The primary endpoint was a composite of in-hospital death, sVT/VF, CS, or AHF. After multiple imputation for missing data, a multi-stage feature selection strategy incorporating least absolute shrinkage and selection operator (LASSO) logistic regression and random forest analysis was applied.

**Results:**

Among 4,897 included patients, 434 (8.9%) experienced the composite endpoint. The final model incorporated 10 readily available clinical variables: age, atrial fibrillation (AF), pulse rate, systolic blood pressure (SBP), neutrophil-to-lymphocyte ratio (NLR), albumin, N-terminal pro-B-type natriuretic peptide (NT-proBNP), estimated glomerular filtration rate (eGFR), glucose, and alanine aminotransferase (ALT). The model demonstrated good discrimination [area under the receiver operating characteristic curve (AUC) 0.808, 95% confidence interval (CI) 0.786–0.830; *p* < 0.001] and satisfactory calibration. The risk score heatmap allows intuitive estimation of individual risk based on cumulative point scores.

**Conclusion:**

We developed an internally validated 10-variable prediction model, visualized as a risk score heatmap, to estimate IAE in older adults with AMI. This intuitive tool may support early bedside risk stratification and personalized management in clinical practice.

## 1 Introduction

Acute myocardial infarction (AMI) remains a leading cause of morbidity and mortality worldwide (1). Despite substantial advances in reperfusion strategies and antithrombotic therapies, early in-hospital complications continue to be major determinants of prognosis, which may also be associated with adverse post-discharge and long-term outcomes, particularly in high-risk populations (2). With the progressive aging of the global population, AMI increasingly affects older adults, who often present with a higher burden of comorbidities, more complex coronary anatomy, and geriatric syndromes such as frailty and polypharmacy. As a result, these patients experience disproportionately higher rates of in-hospital adverse events (IAE) and may derive less benefit from standard therapies in the absence of individualized risk assessment (3). This underscores the critical need for early and accurate risk stratification in this vulnerable population.

Importantly, in-hospital deterioration following AMI extends well beyond mortality alone. Cardiogenic shock (CS) complicates up to 10% of AMI cases and remains the leading cause of in-hospital death (4). In older populations, non-shock complications also account for a substantial share of early clinical deterioration. In a nationwide AMI cohort, heart failure during index hospitalization occurred in 18.7% overall and increased to 25.6–27.1% among patients aged 75–85 years, demonstrating a pronounced age gradient (5). Similarly, malignant ventricular arrhythmias, including sustained ventricular tachycardia or ventricular fibrillation (sVT/VF), although less frequent in contemporary cohorts, remain life-threatening and are strongly associated with early mortality (6). Together, these non-equivalent but complementary complications (pump failure, circulatory collapse, and electrical instability) support the use of a clinically actionable composite endpoint for in-hospital risk assessment. Existing risk stratification tools for AMI often focus on mortality and are not optimized for older, complication-prone patients. While contemporary machine-learning models show promise, their clinical adoption is often hindered by limited interpretability and bedside usability (7).

Therefore, this study aimed to develop and validate a parsimonious, admission-based prediction model for a composite of major IAE—namely death, sVT/VF, CS, and AHF—in older adults with AMI. Critically, we sought to bridge the gap between complex but uninterpretable machine-learning algorithms and overly simplistic traditional scores by translating our model into a novel, risk score heatmap designed for rapid and intuitive bedside application.

## 2 Materials and methods

### 2.1 Study design and patients

This study was a retrospective cohort analysis of patients aged >60 years diagnosed with AMI and admitted to the First Affiliated Hospital of Xi’an Jiaotong University between January 2019 and December 2024. Clinical characteristics, laboratory test results, imaging data, and in-hospital events were obtained from the hospital electronic medical record system and the Biobank of the First Affiliated Hospital of Xi’an Jiaotong University. The study protocol was approved by the Medical Ethics Committee of the First Affiliated Hospital of Xi’an Jiaotong University (XJTU1AF2026LSYY-0109), and informed consent was waived due to the retrospective design.

The inclusion criteria were: (1) age >60 years; and (2) a diagnosis of AMI based on the Fourth Universal Definition of Myocardial Infarction and contemporary ESC acute coronary syndrome guidelines (8, 9). The exclusion criteria were: (1) prior history of chronic liver disease, severe renal dysfunction(defined as pre-existing advanced chronic kidney disease [CKD stage 4–5], end-stage kidney disease, or maintenance hemodialysis), malignancy, or chronic heart failure; (2) comorbid hematologic disorders, autoimmune diseases, or severe psychiatric disorders; (3) hospitalization duration <24 h; (4) insufficient clinical information, defined as extensive missingness in laboratory covariates beyond reliable handling by multiple imputation.

For outcome grouping, patients were classified based on the occurrence of a composite IAE, defined as in-hospital death, sVT/VF, CS, or AHF. In-hospital death was defined as all-cause mortality during the index hospitalization; sVT/VF was defined as documented sustained ventricular tachycardia or ventricular fibrillation requiring medical or electrical intervention; CS and AHF were diagnosed according to contemporary consensus/guideline-based clinical criteria (4, 10, 11). Patients experiencing at least one of these events were assigned to the IAE group, while those without any of these events were assigned to the control group.

### 2.2 Data collection

Baseline demographic characteristics, medical history, laboratory test results, imaging data, and in-hospital events were extracted from the electronic medical record system and the Biobank of the First Affiliated Hospital of Xi’an Jiaotong University. All candidate predictors were derived from data collected at admission or within the first 24 h of hospitalization and were restricted to records documented before the first IAE. Laboratory measurements were derived from the first available tests after admission, comorbidities were identified from documented medical history and discharge diagnoses, and in-hospital outcomes were ascertained from clinical records during the index hospitalization; for patients who developed IAE within 24 h, only measurements documented before the first IAE were used whenever available.

### 2.3 Statistical analysis

After excluding patients with extensive missingness in laboratory covariates, missing data in the retained cohort were handled using multiple imputation by chained equations. The imputation model included all candidate predictors, the composite in-hospital outcome, and auxiliary variables related to missingness/outcome; 20 imputed datasets were generated (m = 20), and estimates were pooled using Rubin’s rules. A detailed comparison of variable missingness and distributions before and after multiple imputation is presented in Supplementary Table 1. The remaining variables had missingness rates below 20%. Continuous variables were standardized to z-scores before regression analyses. The neutrophil-to-lymphocyte ratio (NLR) was calculated as the absolute neutrophil count divided by the absolute lymphocyte count, and the prognostic nutritional index (PNI) was calculated as serum albumin (g/L) + 5 × total lymphocyte count (10^9/L). Estimated glomerular filtration rate (eGFR) was calculated using the Chronic Kidney Disease Epidemiology Collaboration (CKD-EPI) equation.

Continuous variables are presented as mean ± standard deviation or median (interquartile range), as appropriate, and were compared using Student’s t-test or the Mann–Whitney U test. Categorical variables are presented as counts (percentages) and were compared using the chi-square test or Fisher’s exact test. A multi-stage feature selection strategy was employed to identify the most robust and clinically relevant predictors. First, all candidate variables were assessed in univariable logistic regression to initially screen for potential associations. Second, variables with univariable *p* < 0.05 were entered into a least absolute shrinkage and selection operator (LASSO) logistic regression model to reduce dimensionality and mitigate multicollinearity, with the penalty parameter (*λ*) optimized via 10-fold cross-validation. Third, to corroborate variable importance from a non-parametric perspective, variables selected by LASSO were further ranked using a random forest model based on mean decrease in accuracy and mean decrease in Gini index. To improve transparency, model construction was prespecified as follows: (i) start from LASSO-selected variables; (ii) remove treatment-sensitive electrolytes (Na, Cl, Ca, P, and Mg) *a priori*; (iii) evaluate remaining candidates for collinearity/redundancy using random-forest importance and clinical interpretability; and (iv) estimate the final model using multivariable logistic regression. Model performance was assessed using receiver operating characteristic (ROC) curves, calibration curves, and decision curve analysis. Using stratified random sampling based on outcome status, the cohort was randomly divided into a training set (70%) and a validation set (30%). All analyses were conducted using R (version 4.5.2) and SPSS (version 27.0), and a two-sided *p* < 0.05 was considered statistically significant.

## 3 Results

### 3.1 Baseline characteristics and IAE

A total of 4,897 patients aged >60 years with AMI were included in the final analysis. During hospitalization, 434 patients (8.9%) experienced at least one IAE. The incidences of the individual components were 72 (1.5%) in-hospital deaths, 56 (1.1%) sVT/VF events, 69 (1.4%) CS events, and 321 (6.6%) AHF events. Baseline characteristics are summarized in Table 1. Because a single patient could experience more than one component event during hospitalization, these events were not considered mutually exclusive, and the sum of individual components exceeded the number of patients with IAEs.

**Table 1:** Baseline characteristics of older adults with AMI in the IAE and control groups.

Variables	Total (*n* = 4897)	IAE group (*n* = 434)	Control group (*n* = 4463)	*p* value
Age (years)	69 (65, 74)	71 (66, 77)	69(65, 74)	< 0.001
Sex, male (%)	3560 (72.70%)	306 (70.51%)	3254 (72.91%)	0.309
Comorbidities (%)				
Hypertension	2075 (42.37%)	179 (41.24%)	1896 (42.48%)	0.654
Diabetes	1514 (30.92%)	141 (32.49%)	1373 (30.76%)	0.492
CKD	85 (1.74%)	12 (2.76%)	73 (1.64%)	0.127
COPD	78 (1.59%)	10 (2.30%)	68 (1.52%)	0.299
AF	330 (6.74%)	67 (15.44%)	263 (5.89%)	< 0.001
Stroke	736 (15.03%)	61 (14.06%)	675 (15.12%)	0.600
Admission vital signs				
Pulse (bpm)	75 (66, 85)	83 (70, 95)	74 (65, 84)	< 0.001
RR (breaths/min)	19 (18, 20)	20 (18, 20)	19 (18, 20)	< 0.001
SBP (mmHg)	125 (110, 141)	114 (100, 130.75)	126 (110, 142)	< 0.001
DBP (mmHg)	75 (66, 85)	72 (63, 83)	75 (67, 85)	< 0.001
Biochemical results				
PNI	43.65 (39.80, 47.60)	40.12 (36.10, 43.89)	44.00 (40.17, 47.90)	< 0.001
NLR	5.32 (4.07, 7.80)	7.45 (5.01, 12.98)	5.21 (4.02, 7.41)	< 0.001
Hs-CRP (mg/L)	4.34 (1.51, 10.00)	8.67 (2.41, 10.00)	4.14 (1.45, 10.00)	< 0.001
TT (s)	17.20 (16.30, 18.40)	17.20 (16.20, 19.20)	17.20 (16.30, 18.40)	0.429
Fibrinogen (g/L)	3.42 (2.79, 4.32)	3.63 (2.78, 4.71)	3.40 (2.79, 4.28)	0.003
D-dimer (mg/L)	0.57 (0.35, 1.00)	0.97 (0.54, 2.10)	0.54 (0.33, 0.91)	< 0.001
FDP (mg/L)	1.80 (1.21, 2.98)	2.71 (1.65, 5.84)	1.76 (1.20, 2.80)	< 0.001
Neutrophil (%)	76.30 (68.60, 83.40)	81.40 (73.50, 87.77)	75.90 (68.30, 82.90)	< 0.001
Neutrophil (10^9^/L)	7.33 (5.56, 9.61)	8.91 (6.67, 11.85)	7.22 (5.51, 9.42)	< 0.001
Lymphocyte (10^9^/L)	1.28 (0.94, 1.68)	1.13 (0.70, 1.67)	1.29 (0.96, 1.69)	< 0.001
WBC (10^9^/L)	8.23 (6.49, 10.56)	9.98 (7.58, 12.97)	8.10 (6.39, 10.33)	< 0.001
HGB (g/L)	136.00 (124.00, 147.00)	131.00 (118.00, 143.00)	136.00 (124.00, 147.00)	< 0.001
HbA1c (%)	6.00 (5.60, 7.00)	6.10 (5.60, 7.40)	6.00 (5.60, 6.90)	0.050
Glucose (mmol/L)	6.86 (5.51, 9.27)	8.00 (6.14, 12.52)	6.75 (5.47, 9.07)	< 0.001
Lipoprotein(a) (mg/L)	228.00 (126.00, 405.00)	239.00 (139.25, 402.50)	227.00 (125.00, 407.50)	0.346
ApoE (g/L)	33.40 (26.90, 41.80)	33.90 (27.10, 43.48)	33.40 (26.80, 41.50)	0.300
ApoB (g/L)	0.75 (0.62, 0.91)	0.72 (0.58, 0.87)	0.76 (0.63, 0.91)	< 0.001
ApoA (g/L)	1.05 (0.94, 1.19)	1.02 (0.89, 1.17)	1.06 (0.94, 1.19)	< 0.001
LDL (mmol/L)	2.20 (1.70, 2.76)	2.03 (1.54, 2.66)	2.21 (1.72, 2.77)	< 0.001
HDL (mmol/L)	0.94 (0.80, 1.10)	0.95 (0.79, 1.11)	0.94 (0.80, 1.09)	0.829
Triglyceride (mmol/L)	1.16 (0.85, 1.58)	1.04 (0.72, 1.45)	1.18 (0.86, 1.61)	< 0.001
TC (mmol/L)	3.89 (3.27, 4.66)	3.69 (3.07, 4.61)	3.92 (3.29, 4.66)	< 0.001
Hs-cTnT (ng/L)	0.46 (0.10, 1.62)	0.88 (0.19, 2.81)	0.45 (0.10, 1.52)	< 0.001
NT-proBNP (pg/mL)	967.00 (337.20, 2615.00)	2696.00 (683.97, 7017.25)	907.00 (327.90, 2327.00)	< 0.001
CK-MB (U/L)	29.00 (15.00, 89.00)	37.00 (18.00, 118.25)	28.10 (15.00, 86.70)	< 0.001
CK (U/L)	245.00 (99.00, 773.00)	313.00 (115.25, 1156.50)	237.00 (98.00, 750.50)	< 0.001
LDH (U/L)	284.00 (224.00, 424.00)	330.00 (255.25, 582.25)	279.00 (222.00, 411.50)	< 0.001
eGFR (mL/min/1.73 m^2^)	89.90 (75.28, 97.54)	79.95 (55.57, 93.06)	90.48 (76.97, 97.75)	< 0.001
Urea (mmol/L)	5.90 (4.75, 7.35)	6.97 (5.48, 9.29)	5.80 (4.69, 7.21)	< 0.001
Creatinine (μmol/L)	66.00 (55.00, 82.00)	78.50 (64.00, 106.75)	65.00 (54.00, 80.00)	< 0.001
Total Protein (g/L)	62.90 (58.60, 67.60)	61.30 (57.10, 65.70)	63.10 (58.70, 67.80)	< 0.001
Globulin (g/L)	25.90 (23.50, 28.50)	25.60 (23.50, 28.70)	25.90 (23.50, 28.50)	0.372
Albumin (g/L)	37.00 (33.90, 40.00)	34.60 (31.42, 37.55)	37.20 (34.20, 40.20)	< 0.001
AST (U/L)	43.00 (26.00, 97.00)	58.50 (28.00, 170.75)	43.00 (26.00, 94.00)	< 0.001
ALT (U/L)	29.00 (20.00, 44.00)	33.00 (22.00, 59.75)	28.00 (20.00, 43.00)	< 0.001
K (mmol/L)	3.98 (3.70, 4.26)	4.00 (3.62, 4.33)	3.98 (3.70, 4.26)	0.737
Na (mmol/L)	140.00 (137.10, 142.00)	139.00 (136.00, 141.09)	140.00 (137.40, 142.00)	< 0.001
Cl (mmol/L)	102.00 (99.10, 104.90)	101.20 (98.03, 104.27)	102.00 (99.30, 104.95)	< 0.001
Ca (mmol/L)	2.23 (2.12, 2.33)	2.18 (2.06, 2.29)	2.23 (2.13, 2.33)	< 0.001
P (mmol/L)	0.92 (0.77, 1.09)	0.97 (0.79, 1.19)	0.92 (0.76, 1.08)	< 0.001
Mg (mmol/L)	0.99 (0.92, 1.06)	1.00 (0.91, 1.08)	0.99 (0.92, 1.06)	0.248

Data are presented as *n* (%) for categorical variables and median (Q1, Q3) for continuous variables. AMI, acute myocardial infarction; IAE, in-hospital adverse events; CKD, chronic kidney disease; COPD, chronic obstructive pulmonary disease; AF, atrial fibrillation; RR, respiratory rate; SBP, systolic blood pressure; DBP, diastolic blood pressure; PNI, prognostic nutritional index; NLR, neutrophil-to-lymphocyte ratio; hs-CRP, high-sensitivity C-reactive protein; TT, thrombin time; FDP, fibrin degradation products; WBC, white blood cell count; HGB, hemoglobin; HbA1c, hemoglobin A1c; ApoE, apolipoprotein E; ApoB, apolipoprotein B; ApoA, apolipoprotein A1; LDL, low-density lipoprotein; HDL, high-density lipoprotein; TC, total cholesterol; hs-cTnT, high-sensitivity cardiac troponin T; NT-proBNP, N-terminal pro–B-type natriuretic peptide; CK-MB, creatine kinase–MB isoenzyme; CK, creatine kinase; LDH, lactate dehydrogenase; eGFR, estimated glomerular filtration rate; AST, aspartate aminotransferase; ALT, alanine aminotransferase; K, potassium; Na, sodium; Cl, chloride; Ca, calcium; P, phosphate; Mg, magnesium.

Compared with patients without IAE, those who developed IAE were older and more likely to have AF [age: 71.00 (66.00, 77.00) vs. 69.00 (65.00, 74.00), *p* < 0.001; AF: 15.44% vs. 5.89%, *p* < 0.001]. On admission, they showed a less favorable hemodynamic profile, with higher pulse and respiratory rate but lower blood pressure [pulse: 83.00 (70.00, 95.00) vs. 74.00 (65.00, 84.00), *p* < 0.001; respiratory rate: 20.00 (18.00, 20.00) vs. 19.00 (18.00, 20.00), *p* < 0.001; SBP: 114.00 (100.00, 130.75) vs. 126.00 (110.00, 142.00), *p* < 0.001; DBP: 72.00 (63.00, 83.00) vs. 75.00 (67.00, 85.00), *p* < 0.001]. They also had a more adverse inflammatory and organ-function profile, characterized by higher NLR and neutrophil counts, lower lymphocyte counts, higher glucose/NT-proBNP/ALT levels, and lower eGFR and albumin [all *p* < 0.001].

### 3.2 Variable screening and selection

**Figure 1:**
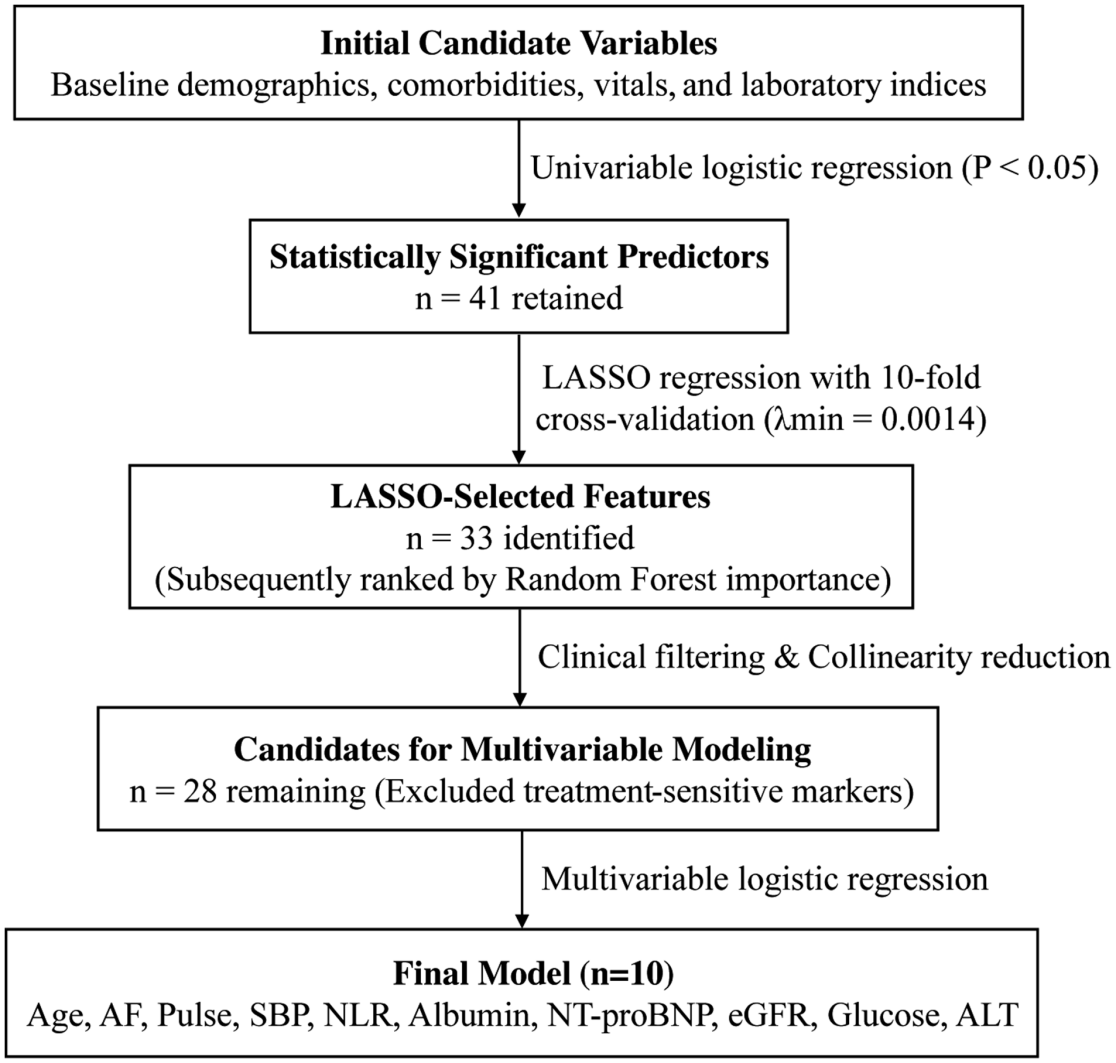
Flowchart of the variable selection process and model construction.

**Figure 2:**
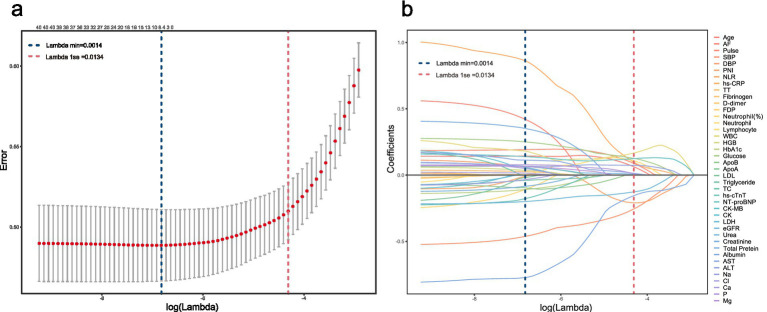
Results of LASSO logistic regression: **(a)** Cross-validation plot for selection of the optimal penalty parameter (*Λ*); **(b)** Coefficient profiles of candidate predictors across log (λ).

**Figure 3:**
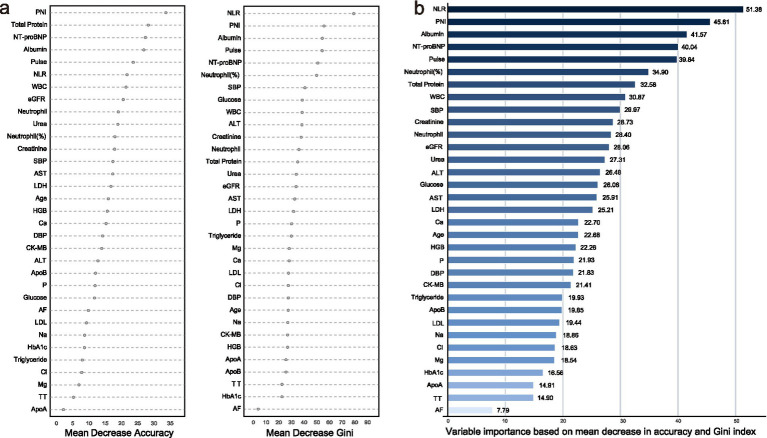
Results of random forest analysis: **(a)** Variable importance ranked by mean decrease in accuracy and mean decrease in Gini; **(b)** Average variable importance based on mean decrease in accuracy and mean decrease in Gini.

The workflow is summarized in Figure 1. After baseline comparisons, univariable logistic regression was used to screen predictors of IAE (Table 2); continuous variables were standardized and odds ratios (ORs) were reported per 1-standard-deviation increase. Variables with *p* < 0.05 were retained (*n* = 41) and entered into LASSO logistic regression with cross-validation; 33 predictors were selected at *λ*.min = 0.0014 (Figure 2). These 33 variables were further ranked using a random forest based on the average of mean decrease in accuracy and mean decrease in Gini (Figure 3), which provided an importance-based prioritization of candidate predictors.

**Table 2:** Univariable logistic regression analysis of candidate predictors; continuous variables were standardized before analysis.

Variables	OR (95% CI)	*p* value
Age (years)	**1.36 (1.24, 1.49)**	***p* < 0.001**
Sex, male (%)	1.13 (0.91, 1.40)	0.283
Comorbidities (%)		
Hypertension	1.05 (0.86, 1.29)	0.618
Diabetes	1.08 (0.88, 1.34)	0.458
CKD	1.71 (0.92, 3.17)	0.089
COPD	1.52 (0.78, 2.98)	0.218
AF	**2.92 (2.18, 3.89)**	***p* < 0.001**
Stroke	0.92 (0.69, 1.22)	0.552
Admission vital signs		
Pulse (bpm)	**1.39 (1.27, 1.53)**	***p* < 0.001**
RR (breaths/min)	1.02 (0.93, 1.12)	0.628
SBP (mmHg)	**0.64 (0.58, 0.70)**	***p* < 0.001**
DBP (mmHg)	**0.76 (0.69, 0.83)**	***p* < 0.001**
Biochemical results		
PNI	**0.50 (0.45, 0.56)**	***p* < 0.001**
NLR	**4.54 (3.57, 5.78)**	***p* < 0.001**
Hs-CRP (mg/L)	**1.42 (1.29, 1.57)**	***p* < 0.001**
TT (s)	**1.14 (1.05, 1.23)**	**0.001**
Fibrinogen (g/L)	**1.20 (1.09, 1.32)**	***p* < 0.001**
D-dimer (mg/L)	**1.15 (1.08, 1.23)**	***p* < 0.001**
FDP (mg/L)	**1.14 (1.07, 1.22)**	***p* < 0.001**
Neutrophil (%)	**1.63 (1.46, 1.82)**	***p* < 0.001**
Neutrophil (10^9^/L)	**1.61 (1.48, 1.76)**	***p* < 0.001**
Lymphocyte (10^9^/L)	**0.80 (0.72, 0.90)**	***p* < 0.001**
WBC (10^9^/L)	**1.70 (1.57, 1.85)**	***p* < 0.001**
HGB (g/L)	**0.77 (0.70, 0.85)**	***p* < 0.001**
HbA1c (%)	**1.10 (1.01, 1.21)**	**0.038**
Glucose (mmol/L)	**1.45 (1.35, 1.57)**	***p* < 0.001**
Lipoprotein(a) (mg/L)	1.01 (0.92, 1.11)	0.825
ApoE (g/L)	1.04 (0.94, 1.14)	0.468
ApoB (g/L)	**0.80 (0.72, 0.89)**	***p* < 0.001**
ApoA (g/L)	**0.82 (0.74, 0.91)**	***p* < 0.001**
LDL (mmol/L)	**0.80 (0.71, 0.89)**	***p* < 0.001**
HDL (mmol/L)	1.00 (0.91, 1.11)	0.933
Triglyceride (mmol/L)	**0.69 (0.60, 0.81)**	***p* < 0.001**
TC (mmol/L)	**0.82 (0.73, 0.91)**	***p* < 0.001**
Hs-cTnT (ng/L)	**1.28 (1.18, 1.38)**	***p* < 0.001**
NT-proBNP (pg/mL)	**1.46 (1.36, 1.55)**	***p* < 0.001**
CK-MB (U/L)	**1.18 (1.09, 1.27)**	***p* < 0.001**
CK (U/L)	**1.22 (1.13, 1.32)**	***p* < 0.001**
LDH (U/L)	**1.41 (1.31, 1.52)**	***p* < 0.001**
eGFR (mL/min/1.73 m^2^)	**0.64 (0.59, 0.69)**	***p* < 0.001**
Urea (mmol/L)	**1.44 (1.34, 1.55)**	***p* < 0.001**
Creatinine (μmol/L)	**1.20 (1.12, 1.28)**	***p* < 0.001**
Total Protein (g/L)	**0.72 (0.65, 0.80)**	***p* < 0.001**
Globulin (g/L)	0.95 (0.86, 1.05)	0.304
Albumin (g/L)	**0.52 (0.47, 0.57)**	***p* < 0.001**
AST (U/L)	**1.40 (1.30, 1.52)**	***p* < 0.001**
ALT (U/L)	**1.40 (1.25, 1.58)**	***p* < 0.001**
K (mmol/L)	1.06 (0.96, 1.17)	0.237
Na (mmol/L)	**0.75 (0.68, 0.82)**	***p* < 0.001**
Cl (mmol/L)	**0.80 (0.73, 0.89)**	***p* < 0.001**
Ca (mmol/L)	**0.72 (0.66, 0.79)**	***p* < 0.001**
P (mmol/L)	**1.30 (1.20, 1.41)**	***p* < 0.001**
Mg (mmol/L)	**1.14 (1.05, 1.24)**	**0.003**

Bold values indicate statistical significance (*P* < 0.05).

OR, Odds ratio; CI, Confidence interval.

### 3.3 Multivariable logistic regression and construction of the final model

**Figure 4:**
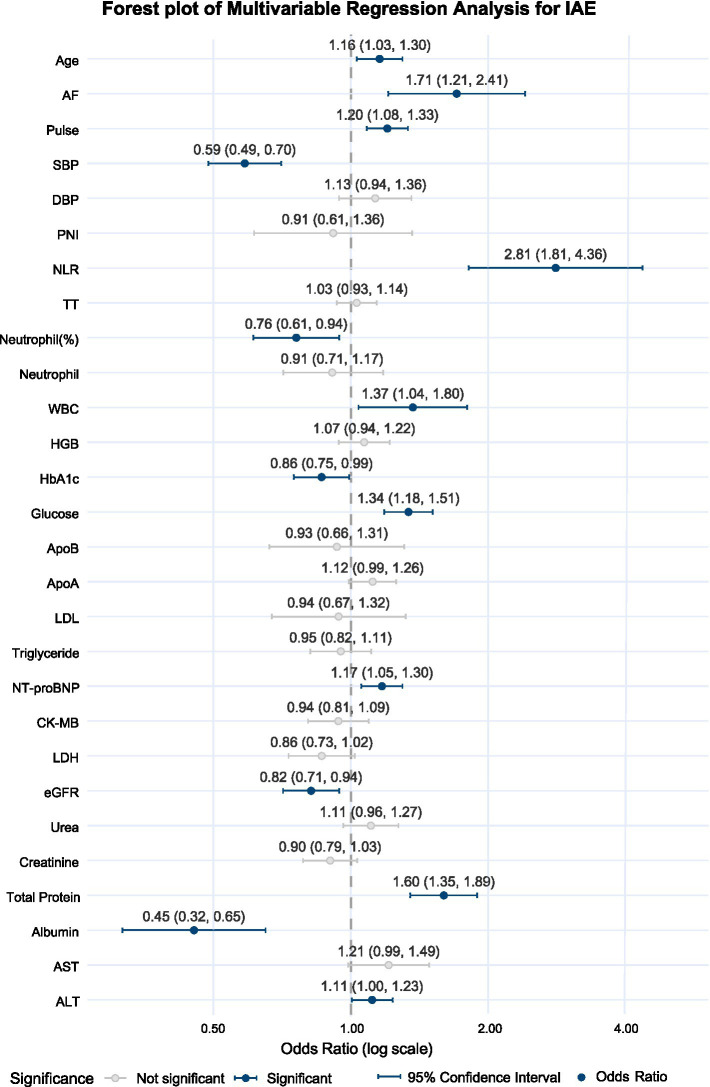
Forest plot of multivariable logistic regression (continuous variables standardized); each horizontal line shows the OR and 95% CI for the corresponding predictor, with continuous variables standardized before modeling. OR, Odds ratio; CI, Confidence interval.

Starting from the 33 LASSO-selected predictors, five treatment-sensitive electrolytes (Na, Cl, Ca, P, and Mg) were excluded *a priori*, leaving 28 candidates for multivariable modeling. We then applied a structured consolidation process: predictors with substantial clinical/statistical overlap were compared using random-forest importance and clinical interpretability, after which multivariable logistic regression was fitted. Variables considered redundant were sequentially removed to obtain a parsimonious final model, while preserving predictive performance and bedside interpretability (Figure 4). Although several predictors remained statistically significant in multivariable analysis—neutrophil percentage, WBC, HbA1c, and total protein—they were not included in the final model to minimize redundancy and improve interpretability. Specifically, neutrophil-related indices and WBC were considered overlapping measures of systemic inflammation and were represented by the clinically integrative NLR; HbA1c was omitted given its collinearity with acute glycemic status captured by glucose; and total protein was excluded due to substantial overlap with albumin, a more clinically interpretable marker of nutritional and inflammatory status.

**Figure 5:**
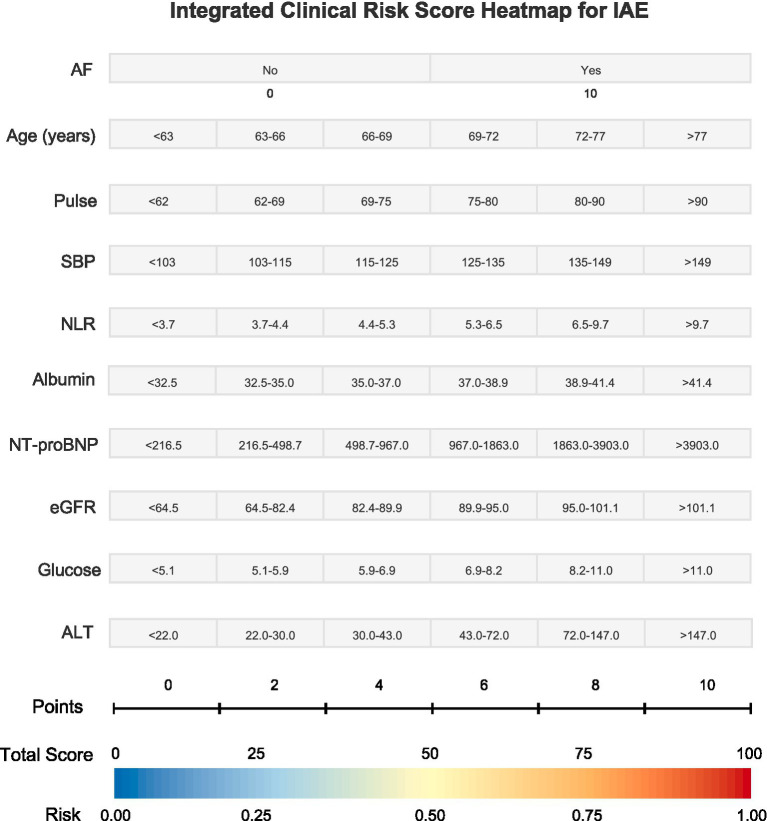
Risk score heatmap for predicting IAE in older adults with AMI, derived from a multivariable logistic regression model.

The final model comprised 10 variables: age, AF, pulse rate, SBP, NLR, albumin, NT-proBNP, eGFR, glucose, and ALT (Figure 5).

### 3.4 Model performance and clinical utility

**Figure 6:**
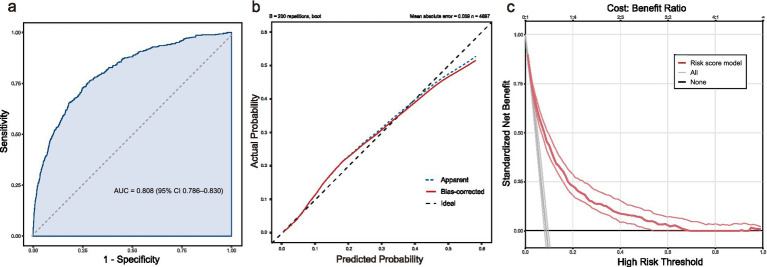
Performance of the risk score heatmap model: **(a)** ROC curve; **(b)** Calibration curve (apparent and bootstrap-corrected); **(c)** Decision curve analysis comparing the model with treat-all and treat-none strategies. ROC, receiver operating characteristic; AUC, area under the ROC curve.

The final model demonstrated good discrimination, with an AUC of 0.808 (95% CI 0.786–0.830) (Figure 6a). Calibration was acceptable, as demonstrated by the calibration curve where the bootstrap-corrected line closely approximated the ideal diagonal line (Figure 6b). The Hosmer–Lemeshow test was non-significant, suggesting no evidence of lack of fit (g = 10 groups: *χ*^2^ = 13.54, df = 8, *p* = 0.095). Decision curve analysis showed that the model provided higher net benefit than both default strategies (“treat-all” and “treat-none”) across a broad and clinically relevant threshold range (approximately 2–66%) (Figure 6c). Notably, within the pragmatic decision range of 5–20%, the model maintained consistent net benefit, supporting its utility for early in-hospital risk-guided management. Decision curve analysis suggested a positive net benefit of the model across a wide range of threshold probabilities, compared with treating all or none (Figure 6c). For internal validation, the model was additionally evaluated in a stratified train/test split (70/30), showing stable discrimination in the training set (AUC = 0.804) and test set (AUC = 0.822) (Supplementary Figure 1). Using the Youden-optimal cutoff derived from the training set (0.079), test-set sensitivity and specificity were 0.762 and 0.741, respectively. To facilitate clinical application, we translated this model into a visual risk score heatmap. Clinicians can calculate a Total Score by summing the points assigned to each variable and then map this score to the bottom axis to directly estimate the patient’s predicted probability of an adverse event. In clinically meaningful subgroups, model discrimination remained acceptable. Subgroup analyses by sex and three baseline comorbidities showed consistently acceptable predictive performance of the model (Supplementary Figure 2).

## 4 Discussion

This study introduces and internally validates a novel, parsimonious risk score for predicting a composite of IAE specifically tailored for older adults with AMI. Our final model incorporates 10 readily accessible variables: age, AF, pulse rate, SBP, NLR, albumin, NT-proBNP, eGFR, glucose, and ALT. Translated into an intuitive risk score heatmap, the model demonstrated good discrimination and acceptable calibration (AUC 0.808, 95% CI 0.786–0.830; Hosmer-Lemeshow *p* = 0.095), with a favorable net benefit across clinically relevant threshold probabilities on decision-curve analysis. Importantly, internal validation using a stratified train/test split showed stable discrimination (AUC 0.804 in training and 0.822 in test), supporting the robustness of the model in this dataset. By providing a visual tool rather than a complex algorithm, we address a critical gap in early risk stratification, offering a simple method with broader clinical coverage than models focused on single endpoints.

A core strength of our model is its integration of fundamental risk factors with dynamic biomarkers. Advancing age, for instance, is a well-established, non-modifiable risk factor for poor outcomes post-AMI, reflecting decreased physiological reserve and a higher burden of comorbidity (12). Similarly, the inclusion of atrial fibrillation, a common arrhythmia in older adults, is consistent with extensive evidence linking it to an increased risk of heart failure, stroke, and mortality in the setting of AMI (13–16). Our findings reaffirm that these fundamental demographic and clinical factors remain cornerstones of risk assessment.

Our study underscores the prognostic relevance of acute hemodynamic instability and systemic inflammation in older adults with AMI. Admission SBP and pulse rate reflect the immediate circulatory response to myocardial injury and neurohumoral activation. Lower SBP at presentation has been consistently associated with worse in-hospital outcomes, including cardiogenic shock and mortality, in patients with AMI (17). Likewise, a higher admission heart rate is a readily available marker of sympathetic overactivation and reduced cardiovascular reserve, and has been linked to increased short-term adverse events and mortality after AMI (18, 19). In parallel, NLR provides an integrated measure of inflammatory activation and immune dysregulation. In ST-segment elevation myocardial infarction (STEMI) cohorts undergoing primary percutaneous coronary intervention (PCI), higher NLR has been associated with impaired myocardial reperfusion/no-reflow and adverse clinical outcomes (20). Moreover, elevated NLR has been shown to predict adverse left ventricular remodeling after STEMI, supporting its relevance beyond the acute phase (21). Contemporary analyses also demonstrate that NLR remains a useful indicator for short-term prognosis and in-hospital death across AMI presentations (22).

Our model also incorporates powerful biomarkers of cardiac and renal stress. NT-proBNP, released in response to myocardial wall stress and volume overload, is one of the most powerful predictors of heart failure development and all-cause mortality following AMI (23, 24). Its high ranking in our analysis underscores the central role of hemodynamic strain in driving in-hospital deterioration. Furthermore, the inclusion of eGFR highlights the profound impact of the cardiorenal syndrome. Chronic kidney disease (CKD) independently accelerates atherosclerosis and is associated with atypical AMI presentations, increased procedural complications, and a markedly higher risk of mortality and heart failure (25). Even mild renal dysfunction, as captured by eGFR, signifies a state of heightened systemic risk (25, 26). The interplay between renal function and cardiac events in AMI patients, especially in the elderly, continues to be a significant area of research (27).

Finally, our model emphasizes the prognostic importance of metabolic and cellular injury markers. Admission hyperglycemia, or “stress hyperglycemia,” is a potent predictor of adverse outcomes in AMI, irrespective of a prior diagnosis of diabetes mellitus. In the present model, the inclusion of ALT rather than AST should be interpreted as a data-driven variable selection result aimed at maximizing overall prognostic performance and bedside interpretability, rather than as evidence that AST is biologically unimportant in AMI (28–30). In older AMI patients, AST may be less specific because it is released not only from myocardium and liver but also from skeletal muscle and erythrocytes, and can be influenced by concomitant muscle injury, hemolysis, or hepatic congestion. By contrast, ALT is more hepatocyte-predominant and may better capture liver hypoperfusion/congestion during early hemodynamic compromise, a clinically meaningful signal of systemic low-flow status. This interpretation is aligned with evidence showing that liver-injury signals, including ALT elevation, are associated with worse outcomes in cardiogenic shock and heart failure populations beyond AMI- only cohorts (31).

The primary novelty of our study is the translation of the statistical model into a visually intuitive risk score heatmap. Although numerous AMI risk scores have been proposed, many are limited by complexity, mortality-centered scope, or suboptimal bedside usability. While contemporary prediction approaches, including machine-learning models, may achieve high accuracy, limited interpretability remains a major barrier to routine clinical implementation (7). In our heatmap, continuous predictors were discretized into ordered ranges using data-driven cut points derived from the development cohort (presented as six strata in Figure 5). Points were then assigned through linear transformation of the final multivariable logistic-regression coefficients, with progressively higher points for higher-risk strata; binary variables (e.g., AF) were scored directly according to model coefficients. This framework enables bedside application by selecting the appropriate stratum for each predictor, summing the points, and mapping the total score to the predicted event probability.

Our study has several limitations. First, as a retrospective, single-center study, our findings are subject to inherent design-related limitations, including potential selection bias and unmeasured confounding, and their generalizability to other populations or healthcare systems requires further confirmation. Second, the model was intentionally constructed using admission-based variables to enable early risk stratification; consequently, in-hospital treatment details (e.g., reperfusion strategies), which may act as downstream mediators of outcomes, were not incorporated. Third, although multiple imputation was applied to address missing data, the possibility of residual bias cannot be entirely excluded. In addition, the study period spanned several years during which updates to AMI management guidelines occurred, which may have introduced temporal heterogeneity in clinical practice. Finally, the model has undergone internal validation only; therefore, its performance, robustness, and clinical utility require prospective, multicenter external validation before consideration for widespread clinical adoption. In addition, direct head-to-head comparison with established AMI risk instruments (e.g., GRACE/TIMI) was not performed in the current dataset and should be addressed in future external studies.

## 5 Conclusion

In this study, we developed and internally validated a novel 10-variable risk score heatmap for predicting composite IAE in older adults with acute myocardial infarction. By integrating readily accessible parameters that reflect the patient’s underlying inflammatory, nutritional, and metabolic state, this tool provides a simple yet comprehensive framework for early risk stratification. The visual, point-based format is designed to facilitate rapid bedside decision-making, potentially helping clinicians identify high-risk individuals who may warrant more intensive monitoring or tailored therapeutic interventions. While promising, rigorous external validation is an essential next step before this tool can be considered for widespread clinical implementation. Ultimately, this risk score heatmap represents a tangible step toward more personalized and proactive care for this high-risk patient population.

## Data Availability

The raw data supporting the conclusions of this article will be made available by the authors, without undue reservation.
