# Supplementary material for: Development and validation of a risk score heatmap for in-hospital adverse events in older adults with acute myocardial infarction
Source: Front Med (Lausanne). 2026 May 4;13:1828310. doi: 10.3389/fmed.2026.1828310 (PMC13195306; doi:10.3389/fmed.2026.1828310)

Supplementary Table 1. Comparison of Variable Missingness and Distribution Before and After Multiple Imputation

| **Variable** | **Missing, %** | **Median before imputation** | **Median after imputation** | **Mean before imputation** | **Mean after imputation** |
| --- | --- | --- | --- | --- | --- |
| PNI | 9.52 | 43.75 | 43.65 | 43.96 | 43.81 |
| NLR | 4.68 | 5.34 | 5.32 | 7.26 | 7.32 |
| Hs-CRP (mg/L) | 17.98 | 4.37 | 4.34 | 5.29 | 5.27 |
| TT (s) | 7.39 | 17.20 | 17.20 | 26.73 | 26.56 |
| Fibrinogen (g/L) | 7.39 | 3.41 | 3.42 | 3.60 | 3.61 |
| D-dimer (mg/L) | 7.88 | 0.58 | 0.57 | 1.39 | 1.37 |
| FDP (mg/L) | 16.64 | 1.83 | 1.80 | 4.64 | 4.48 |
| Neutrophil (%) | 4.68 | 76.30 | 76.30 | 75.55 | 75.56 |
| Neutrophil (10⁹/L) | 4.68 | 7.14 | 7.33 | 7.86 | 7.95 |
| Lymphocyte (10⁹/L) | 4.68 | 1.28 | 1.28 | 1.39 | 1.37 |
| WBC (10⁹/L) | 4.68 | 8.21 | 8.23 | 8.83 | 8.85 |
| HGB (g/L) | 4.68 | 135.00 | 136.00 | 134.26 | 134.58 |
| HbA1c (%) | 12.03 | 6.00 | 6.00 | 6.55 | 6.55 |
| Glucose (mmol/L) | 4.84 | 6.86 | 6.86 | 8.11 | 8.10 |
| Lipoprotein(a) (mg/L) | 10.09 | 230.00 | 228.00 | 317.00 | 317.06 |
| ApoE (g/L) | 10.09 | 33.20 | 33.40 | 35.75 | 35.91 |
| ApoB (g/L) | 10.09 | 0.75 | 0.75 | 0.77 | 0.77 |
| ApoA (g/L) | 10.09 | 1.06 | 1.05 | 1.07 | 1.07 |
| LDL (mmol/L) | 9.37 | 2.20 | 2.20 | 2.30 | 2.29 |
| HDL (mmol/L) | 9.37 | 0.95 | 0.94 | 0.97 | 0.97 |
| Triglyceride (mmol/L) | 9.35 | 1.14 | 1.16 | 1.31 | 1.33 |
| TC (mmol/L) | 6.29 | 3.89 | 3.89 | 4.02 | 4.02 |
| Hs-cTnT (ng/L) | 16.18 | 0.41 | 0.46 | 1.32 | 1.39 |
| NT-proBNP (pg/mL) | 9.21 | 967.90 | 967.00 | 2528.56 | 2526.45 |
| CK-MB (U/L) | 6.06 | 28.00 | 29.00 | 77.86 | 78.46 |
| CK (U/L) | 4.82 | 240.00 | 245.00 | 727.32 | 725.48 |
| LDH (U/L) | 4.86 | 284.00 | 284.00 | 378.94 | 378.59 |
| eGFR (mL/min/1.73 m²) | 12.51 | 89.58 | 89.90 | 84.85 | 84.18 |
| Urea (mmol/L) | 4.68 | 5.91 | 5.90 | 6.51 | 6.50 |
| Creatinine (μmol/L) | 4.68 | 66.00 | 66.00 | 76.96 | 76.89 |
| Total Protein (g/L) | 9.54 | 62.80 | 62.90 | 63.13 | 63.18 |
| Globulin (g/L) | 9.54 | 26.00 | 25.90 | 26.17 | 26.12 |
| Albumin (g/L) | 9.43 | 36.90 | 37.00 | 36.99 | 36.98 |
| AST (U/L) | 6.58 | 43.00 | 43.00 | 90.27 | 89.63 |
| ALT (U/L) | 9.54 | 28.00 | 29.00 | 41.44 | 41.44 |
| K (mmol/L) | 4.66 | 3.98 | 3.98 | 3.99 | 3.99 |
| Na (mmol/L) | 4.76 | 140.00 | 140.00 | 139.56 | 139.56 |
| Cl (mmol/L) | 4.76 | 102.00 | 102.00 | 101.98 | 101.94 |
| Ca (mmol/L) | 4.76 | 2.23 | 2.23 | 2.22 | 2.22 |
| P (mmol/L) | 4.76 | 0.92 | 0.92 | 0.95 | 0.95 |
| Mg (mmol/L) | 4.70 | 0.99 | 0.99 | 1.00 | 1.00 |

Notes: Missingness is presented as percentage before imputation. Means and medians are shown before and after multiple imputation by chained equations (MICE). Values are reported to two decimal places.

Supplementary Figure 1. ROC Curves in Training and Validation Sets.


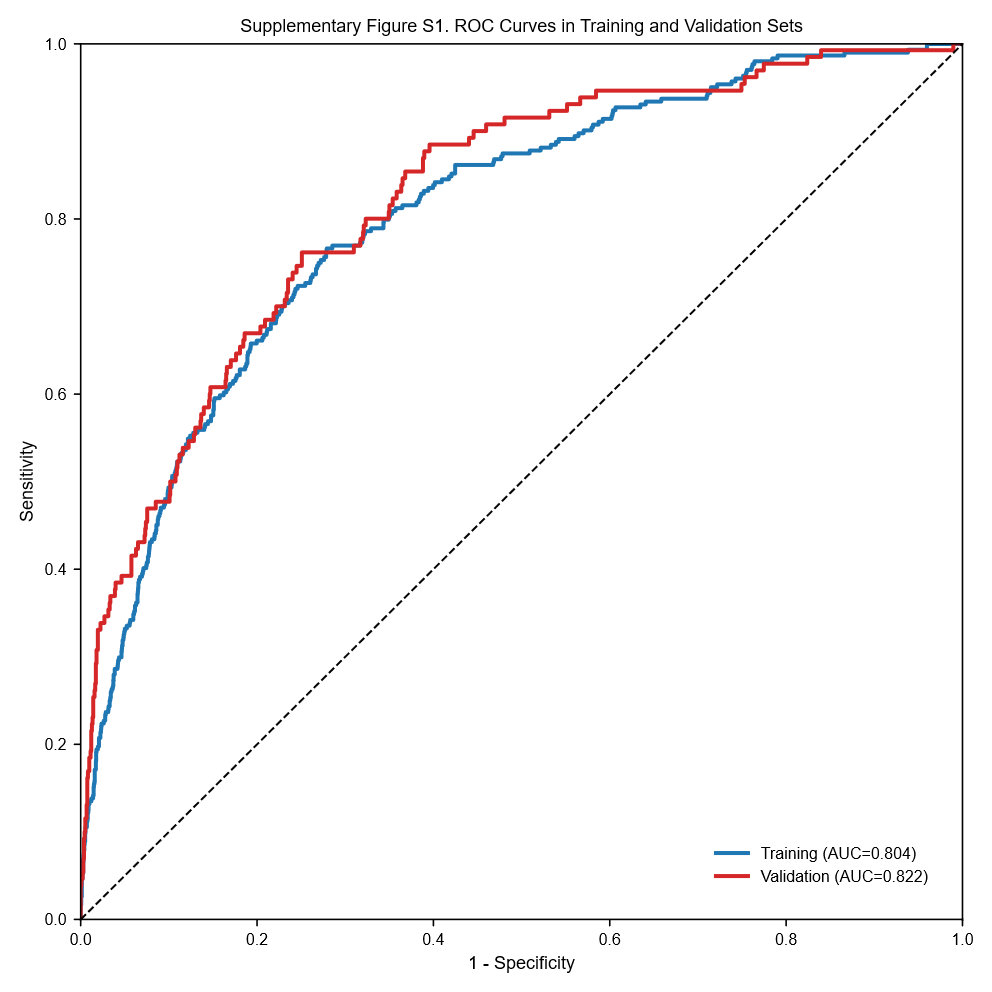


Supplementary Figure 2. Subgroup ROC Curves by Sex and Comorbidities.


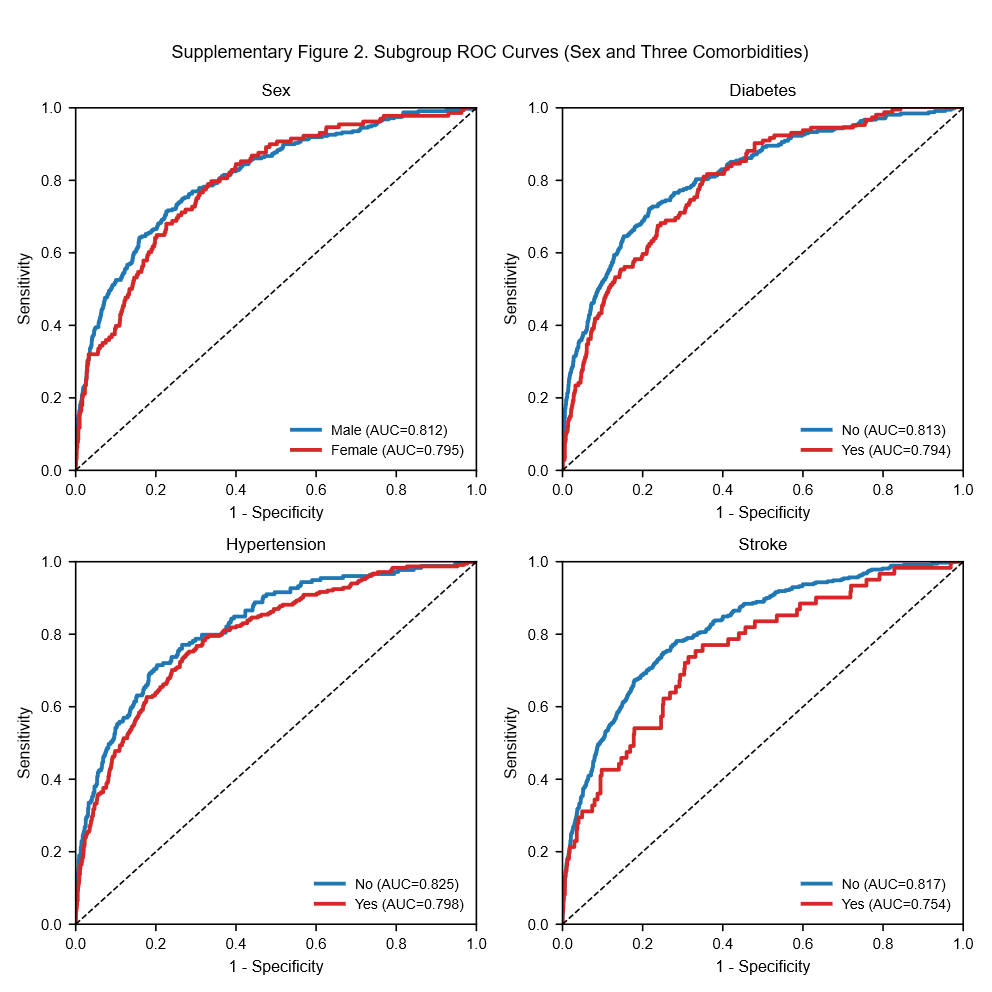

Supplement: Supplementary file 1 [file Data_Sheet_1.docx]
